# Epidemiology of Neonatal Sepsis and Implicated Pathogens: A Study from Egypt

**DOI:** 10.1155/2015/509484

**Published:** 2015-06-04

**Authors:** Eman M. Rabie Shehab El-Din, Mohamed M. Adel El-Sokkary, Mohamed Reda Bassiouny, Ramadan Hassan

**Affiliations:** ^1^Unit of Drug Analysis, Mansoura University, Mansoura 35516, Egypt; ^2^Department of Microbiology and Immunology, Faculty of Pharmacy, Mansoura University, Mansoura 35516, Egypt; ^3^Department of Pediatrics, Faculty of Medicine, Mansoura University, Mansoura 35516, Egypt

## Abstract

Prospective analytic study was conducted in NICUs of three Egyptian Neonatal Network (EGNN) participants in Mansoura Hospitals in Egypt over a period of 18 months from March 2011 to August 2012. By using EGNN 28-day discharge form, all demographic, clinical, and laboratory data were recorded and studied. During the study period, 357 neonates were diagnosed as suspected sepsis with an incidence of 45.9% (357/778) among the admitted neonates at the three neonatal intensive care units. 344 neonates (sex ratio = 1.3:1) were enrolled in the study in which 152 (44.2%) were classified as early onset sepsis EOS (≤72 hr) and 192 (55.8%) as late onset sepsis LOS (>72 hr). Among the LOS cases, 33.9% (65/192) were caused by nosocomial infections. In 40.7% (140/344), sepsis was confirmed by positive blood culture. The total mortality rate for the proven neonatal sepsis was 51% (25/49) and 42.9% (39/91) for EOS and LOS, respectively. Coagulase negative staphylococci were predominant isolates in both EOS and LOS, followed by *Klebsiella pneumoniae*. Most of the bacterial isolates had low sensitivity to the commonly used empiric antibiotics. However, 70.1% (89/127) exhibited multidrug resistance. Best sensitivities among Gram-positive isolates were found against imipenem, ciprofloxacin, vancomycin, and amikacin.

## 1. Introduction

Globally, sepsis is still one of the major causes of morbidity and mortality in neonates, in spite of recent advances in health care units [1]. More than 40% of under-five deaths globally occur in the neonatal period, resulting in 3.1 million newborn deaths each year [2]. The majority of these deaths usually occur in low-income countries and almost 1 million of these deaths are attributed to infectious causes including neonatal sepsis, meningitis, and pneumonia [3]. On the other hand, the survivors of neonatal sepsis are vulnerable to short- and long-term neurodevelopmental morbidity [4–6].

Neonatal sepsis is defined as a clinical syndrome in an infant 28 days of life or younger, manifested by systemic signs of infection and isolation of a bacterial pathogen from the bloodstream [7]. Diagnosis and management of sepsis are a great challenge facing neonatologists in NICUs. Clinical diagnosis of presentation is difficult due to nonspecific signs and symptoms. In addition, laboratory diagnosis is time consuming. This matter necessitates the initiation of empirical antibiotic therapy till the suspected sepsis is ruled out. At the same time, increased multidrug resistant organisms make the treatment options fewer and the effective treatment is delayed [8].

Neonatal sepsis is caused by Gram-positive and Gram-negative bacteria and* Candida* [9]. The diversity of organisms causing sepsis varies from region to another and changes over time even in the same place [10, 11]. This is attributed to the changing pattern of antibiotic use and changes in lifestyle. Many factors contribute to the susceptibility of the neonate to sepsis, which can influence the incidence of neonatal sepsis. Incidence also varies from nursery to nursery depending on conditions predisposing infants to infection [9, 12].

The aim of the present study was to evaluate the incidence of neonatal sepsis and characterize the microbiological pattern of neonatal sepsis and the antibiotic susceptibility of the isolates to evaluate the empirical antibiotic used in neonatal units of three referral hospitals in Mansoura, Egypt.

## 2. Materials and Methods

### 2.1. Study Design and Population

This study was prospectively conducted over a period of 18 months between March 2011 and August 2012, at three NICUs in Mansoura City, Egypt, namely, Mansoura University Children Hospital (MUCH), Health Insurance Hospital (HIH), and Mansoura General Hospital (MGH). During the study period, all admitted neonates with clinical signs and symptoms of sepsis at the time of admission or who developed sepsis during their hospital stay were assessed using EGNN sepsis screening tool and included in the study.

### 2.2. Patient Data

Using EGNN guidelines, a standard structured data collection form was designed to obtain social demographic, clinical, and laboratory data that were recorded by qualified medical staff. All neonates were subjected to full clinical examination stressing on gestational age, birth weight, mode of delivery, and risk factors for sepsis: premature rupture of membranes (PROM), maternal fever, insertion of an umbilical catheter, and so forth.

According to the Egyptian Neonatal Network (EGNN), sepsis is defined as presence of at least 3 out of the following four criteria [13]:

- presence of risk factors of sepsis (e.g., prematurity, chorioamnionitis),
- presence of two or more clinical signs of sepsis (poor reflexes, lethargy, respiratory distress, bradycardia, apnea, convulsions, abdominal distension, and bleeding),
- abnormal hemogram and positive CRP and positive culture.

Patient receives antibiotics and antifungal for at least five days (or <5 days if he is transferred or died before completion of these five days).

According to the infant age, at the onset of symptoms, neonates were classified into two groups:* EoNS* (≤72 hours of life) and* LoNS* (>72 hours of life) [14].

### 2.3. Nosocomial Infection

It was defined by Standard Center for Disease Control and Prevention [15] as an infection acquired during hospitalization (>48 hrs) and resulted from an organism inoculation that was not present in the patient at the time of admission [15, 16], excluding cases of early-onset sepsis.

### 2.4. Collection of Specimens

Blood samples were collected from the neonates with suspected sepsis for CRP, CBC, and blood cultures. Blood was collected from a peripheral vein. Approximately 1 mL of blood was inoculated directly into blood culture medium vials and sent to our clinical microbiology laboratory for cultivation and subsequent processing.

### 2.5. Processing of Specimens

The blood cultures were incubated aerobically at 37°C and observed daily for the first 3 days for the presence of visible microbial growth by one of the following: haemolysis, air bubbles (gas production), and coagulation of broth. At the same time, subcultures were made during 3 successive days on enriched and selective media including blood, chocolate, MacConkey, and mannitol salt agar plates and examined for growth after 24–48 hours of incubation. The same protocol was repeated until the 7th day before blood culture was considered to be free of microorganisms. Isolates obtained were identified by standard microbiological techniques, namely, Gram staining, colony characteristics, and biochemical properties including catalase, coagulase (free and bound), DNase production, growth on mannitol salt agar, and hemolytic activity on blood agar plates for Gram-positive isolates, and triple sugar iron (TSI), motility, indole, citrate utilization, urease, oxidase and hydrogen sulphide production, Voges-Proskauer (VP) test, and growth on cetrimide agar for Gram-negative bacilli [17]. API 20E identification kits (bioMe rieux) were also used to confirm the identification of Gram-negative isolates and the results were read using API 32 GN reader. Candida isolates were confirmed by growth on Sabouraud media.

### 2.6. Identification of* Staphylococci* Species

*Staphylococcus* species were identified by PCR-Restriction Fragment Length Polymorphism of* gap* Gene, using* Alu*I as restriction enzyme, resulting in a distinctive RFLP pattern for every species as previously described [18].

### 2.7. Antimicrobial Susceptibility Testing

Antimicrobial susceptibility testing of all bacterial isolates was performed by the Kirby-Bauer disc diffusion method on Mueller-Hinton agar (Oxoid) according to the recommendations of the CLSI (2010). The antibiotics tested were ampicillin (10 *μ*g), oxacillin (1 *μ*g), amoxicillin-clavulanic acid (30 *μ*g), cefoxitin (30 *μ*g), cefotaxime (30 *μ*g), ceftriaxone (30 *μ*g), ceftazidime (30 *μ*g), imipenem (10 *μ*g), vancomycin (30 *μ*g), gentamicin (10 *μ*g), amikacin (30 *μ*g), erythromycin (15 *μ*g), azithromycin (15 *μ*g), ciprofloxacin (5 *μ*g), and norfloxacin (10 *μ*g).

*Multidrug Resistant (MDR) Bacteria.* They were defined by resistance to three or more antimicrobial classes [19].

### 2.8. Statistical Analysis

Summary of measures was reported as mean ± standard deviation (SD) for quantitative variables and percentages for categorical variables. The differences in distribution were evaluated using the chi-square test for categorical variables. *P* value ≤ 0.05 was considered statistically significant. All the statistical analyses were performed using GraphPad InStat version 3.05.

## 3. Results

### 3.1. Studied Population

During the study period, a total of 357 neonates with suspected cases of sepsis were enrolled. Thirteen cases were excluded: eleven blood cultures were lost and the other two cultures were contaminated. As a result, the final number subjected for the report was 344. The incidence of suspected neonatal sepsis among the admitted neonates at the neonatal intensive care units of the three included hospitals during the study period was 45.9% (357/778). Among the studied neonates, sepsis was recognized as EOS in 152 (44.2%) cases and as LOS in 192 (55.8%) cases according to infant age at the onset of symptoms. 33.9% (65/192) of LOS were due to nosocomial infection (Table 1).

The sepsis was proved in 140 (40.7%) cases by positive blood culture: 49 from early-onset and 91 from late-onset sepsis. There was a significant difference in the positivity rate between EOS and LOS groups (*P* < 0.05).

Among the studied neonates, 195 (56.7%) were males and 149 (43.3%) were females resulting in an overall male to female ratio of 1.3 : 1. However, no significant difference was detected with regard to sex (*P* > 0.05). The total mortality rate for the confirmed neonatal sepsis was estimated as 51% for EOS and 42.9% for LOS.

### 3.2. Maternal and Neonatal Characteristics and Clinical Features

Maternal and demographic data and clinical information were available for 304 patients (88.4%) as shown in Tables 2 and 3. Of the 304 neonates, 179 (58.9%) were preterm. 296 (97.4%) were born in the health care facilities (hospitals/clinics) and 8 (2.6%) were born at home. Referring to the delivery mode, 212 (69.7%) were delivered by caesarean section whereas 92 (30.3%) were delivered vaginally. Approximately, 212 (69.7%) neonates with sepsis had low birth weight (<2500 g), and out of these 81 (38.2%) had very low birth weight (<1500 g). The mean gestational age and birth weight of the study population were 34.4 ± 3.8 weeks and 2124 ± 828 grams, respectively. The most prevalent clinical feature was respiratory distress (41.3%).

### 3.3. Other Investigations

#### 3.3.1. C-Reactive Protein Results

Among the 344 neonates admitted with suspected cases of sepsis, the CRP level was measured in 326 cases where it was positive (>6 mg/L) in 278 (85.3%) cases.

#### 3.3.2. Hematological Parameters (White Blood Cell and Platelet Counts)

CBC was determined in 319 cases. Abnormalities in the CBC were found in 213 (66.8%) neonates with 22 (6.9%) having leucopenia (WBC < 5,000/mm^3^), 71 (22.3%) leukocytosis (WBC > 20,000/mm^3^), 74 (23.2%) neutropenia, and 145 (45.5%) thrombocytopenia (platelets < 140,000/mm^3^).

#### 3.3.3. Isolated Pathogens

**Figure 1:**
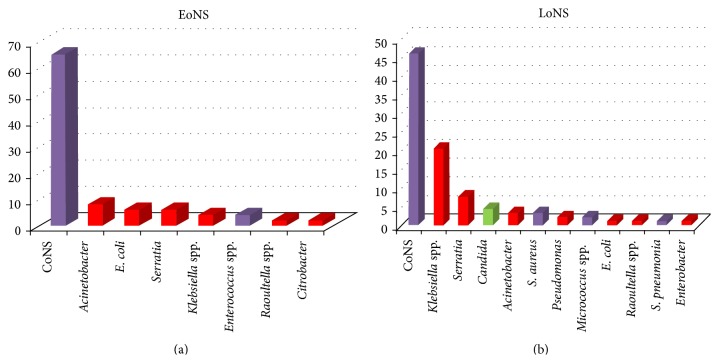
Microbiological profile found in positive blood cultures from neonates with EoNS (a) and LoNS (b).

Out of the 344 blood cultures, only 140 (40.7%) showed growth of different bacteria and fungi. The type and frequency of isolated pathogen in relation to the type of sepsis were shown in Table 4 and Figure 1. Gram-positive bacteria were responsible for most cases of neonatal sepsis. Coagulase negative staphylococci (CoNS) were the most frequent isolated pathogens in EoNS and LoNS, followed by* Klebsiella pneumoniae *and* Serratia marcescens*.

#### 3.3.4. Antibiotic Susceptibility Pattern

**Figure 2:**
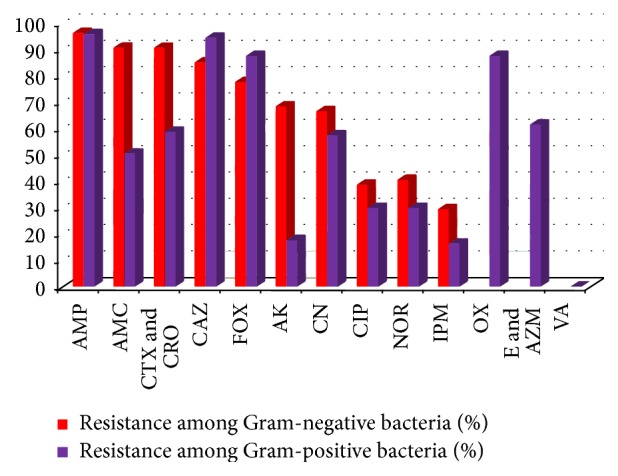
Comparative percentage of resistance to the tested antimicrobial agents among Gram-negative isolates and Gram-positive isolates.

**Figure 3:**
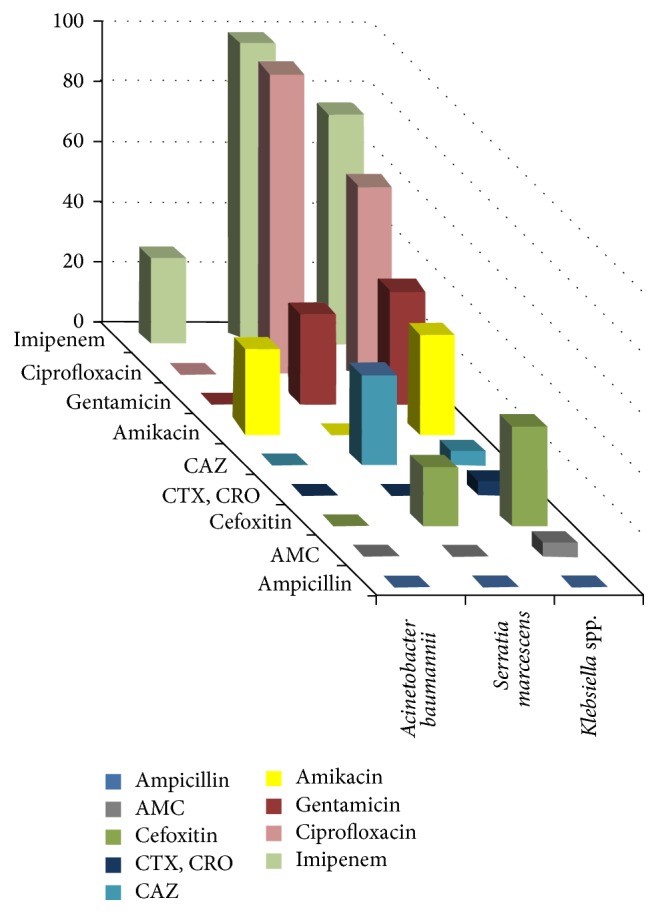
Comparative sensitivities of different Gram-negative bacteria to different antimicrobial agents.

**Figure 4:**
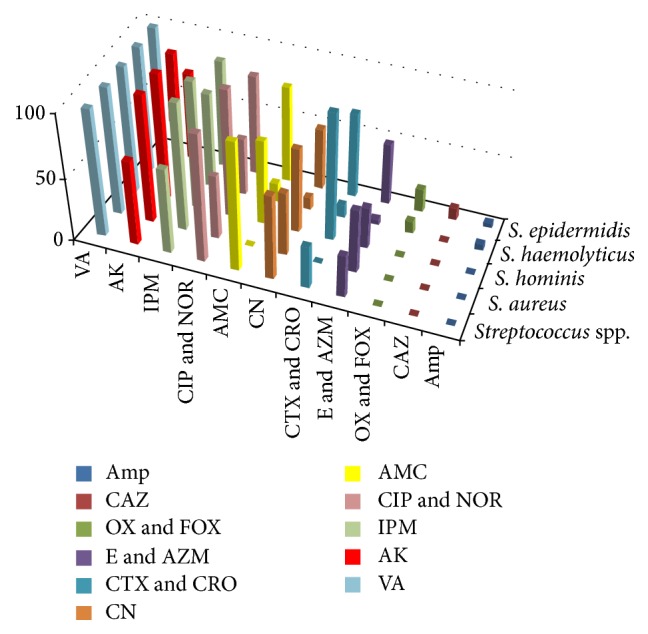
Comparative sensitivities of different Gram-positive bacteria to different antimicrobial agents.

The sensitivity patterns of the bacterial isolates to first- and second-line empiric antibiotics commonly used in neonatal infection were illustrated in Tables 5 and 6 and Figures 2, 3, and 4. Quinolones (ciprofloxacin) are not recommended for use in young children, but they may be used in culture-proven sepsis with bacteria resistant to other antibiotics.

*Gram-Positive Bacteria.* They showed high resistance to ampicillin (95.9%). The intermediate effect was observed (50.7%) with amoxicillin-clavulanic acid. In contrast to gentamicin, amikacin was highly effective on Gram-positive isolates. Best sensitivity was also observed to imipenem and ciprofloxacin. All isolates were sensitive to vancomycin. Among the* Staphylococci* spp.,* S. haemolyticus* isolates were highly resistant.

*Gram-Negative Bacteria.* They were highly resistant to the first- and second-line empiric antibiotics: ampicillin (96.3%), amoxicillin-clavulanic acid (90.7%), gentamicin (66.7%) and amikacin (68.5%), and 3rd generation cephalosporins (>85%). Best sensitivity was observed to imipenem and ciprofloxacin. The effect of the tested antimicrobial agents was variable according to the genus as illustrated in Table 6 and Figure 3. It was found that imipenem and ciprofloxacin had a strong effect on* Serratia* isolates followed by* Klebsiella* isolates, whereas* Acinetobacter* isolates were resistant to all antimicrobial agents except imipenem and amikacin on a small number.

#### 3.3.5. Multidrug Resistance (MDR)

MDR was observed in 89 isolates (70.1%). Among the Gram-positive isolates, 53.4% (39/73) were multidrug resistant while, among Gram-negative isolates, MDR was detected in 92.6% (50/54).

## 4. Discussion

The clinical signs and symptoms of neonatal sepsis are subtle and nonspecific, making its early diagnosis difficult, and it can interfere with other life-threatening diseases, such as necrotizing enterocolitis and perinatal asphyxia [20, 21]. Blood culture is still the gold standard for definitive diagnosis of neonatal sepsis, in spite of some drawbacks of blood cultures as being time consuming, low sensitivity, and possible contamination especially with commensal CoNS that could be produced.

In our study, the incidence of suspected neonatal sepsis during the study period was 45.9% with a mortality rate of 51% for proven EOS and 42.9% for proven LOS. Similar high rates were previously reported in Egypt [22] and other developing countries such as Tanzania 39% [23] and Cameroon 34.7% [24]. In contrast, very low rates were reported in the developed countries [25], which can be explained by the high quality of life and high standard measures of health care and hospital services in these countries.

During the study period, 344 neonates with suspected neonatal sepsis (using clinical criteria) were enrolled. Only 140 (40.7%) were confirmed to have bloodstream infection by using blood culture. This rate is comparable to rates reported in other developing African and Asian countries as Bangladesh (34.88%) [26], Uganda (37%) [27], Ethiopia (44.7%) [28], and Nigeria (45.9%) [29]. However, negative blood culture does not exclude sepsis as about 26% of all neonatal sepsis could be due to anaerobes [30]. Furthermore, the etiological agent may not be isolated by media used in our study such as viral (e.g., rubella, cytomegalovirus), protozoal (e.g.,* Toxoplasma gondii*), and treponemal (e.g.,* Treponema pallidum*) pathogens.

Among the studied neonates, LOS (55.8%) was more common than EOS (44.2%), which is in agreement with reports from other African and Asian countries [23, 31, 32]. However, the opposite was documented in some previous reports [33–35].

In our study, the incidence of sepsis was higher in neonates born via CS than in those born via VD. This finding is similar to other previous studies [33, 36, 37]. For example, in the study of Utomo et al. (2010), in Indonesia (Surabaya), it was reported that infants delivered via CS have 1.89 times higher risk to develop sepsis than noncaesarean.

In our study, the incidence of neonatal sepsis in both EOS and LOS was predominantly associated with Gram-positive cocci, specifically CoNS compared to Gram-negative and* Candida *spp. Similar findings were obtained in other studies in Egypt [38] and other different countries (including China, Mexico, South Africa, and Kenya) [25, 31, 39–41]. High rates of CoNS infections were reported in the Middle East, Southeast Asia, and Latin America [42]. In some studies, CoNS were more common to cause LOS. However, true EOS caused by CoNS was proved in other studies [43, 44]. On the contrary, Gram-negative neonatal sepsis was predominant in other studies [24, 28, 33–35, 45–48].

The extensive use of invasive devices for caring for the immunologically immature neonates especially preterm and LBW is the main cause of CoNS bacteremia in NICU. This finding is supported by the study of Kerur et al., as, in more than 50% of the cases of CoNS bacteremia in NICU, the infection could be correlated with the use of venous catheters.

Despite the importance and role of CoNS as etiological agents of neonatal sepsis as proved in many studies, determination of the identity of CoNS isolates whether being true pathogens or contaminants is still problematic. In our study,* S. epidermidis *was the most frequently recovered CoNS isolate in blood cultures, followed by* S. haemolyticus*. These two species were present at 55% and 33.3% in blood cultures, respectively. Similar findings were reported in other previous studies [25, 49, 50].

Gram-negative bacteria were the 2nd cause of neonatal sepsis especially LOS following CoNS, with increased mortality rate.* Klebsiella *spp. (15%), mainly* K. pneumoniae*, were the most predominant Gram-negative pathogen, followed by* Serratia marcescens* (7.14%) and* Acinetobacter baumannii* (5%). In our study,* Klebsiella* isolates were responsible for 4.08% and 20.88% of EOS and LOS, respectively. Other Gram-negative bacilli were recovered but in a few numbers. The predominance of* Klebsiella* among the causative Gram-negative pathogens was also reported in other studies in Egypt [45, 48] and other different countries [24, 25, 39, 40, 46, 51]. On the contrary, other Gram-negative bacteria such as* E. coli* [35, 47, 52],* P. aeruginosa* [34, 53], and* Enterobacter *spp. [33] could be identified as the most common Gram-negative isolates associated with neonatal sepsis.

*Serratia marcescens was *the 2nd higher Gram-negative isolate. This was in consistency with a study in Bangladesh in which* S. marcescens* was the 2nd Gram-negative isolate following* K. pneumoniae* with a rate of 18.27% [54]. Moreover, in another study in Europe,* S. marcescens* caused 5% of neonatal bloodstream infections in NICU [55]. However, in other studies* S. marcescens *could not be detected among pathogens isolated from cases of neonatal sepsis [25, 31, 39]. In the last decade, several outbreaks in NICU were documented [56–58], causing potentially fatal sepsis, meningitis, or pneumonitis in very premature or low birth weight neonates with high mortality rate.

*Acinetobacter baumannii* was isolated in our study from 5% of positive blood cultures of septic neonates accounting for 8.16% and 3.30% in EOS and LOS, respectively. Similarly, septic neonates infection ranging from 3.5% up to 7.7% has been previously recognized [24, 35, 36, 42, 46].

*Candida *spp. were isolated only in 4 cases (2.86%) causing LOS; two were born preterm, a known risk factor for candidemia [59]. Similar findings were found in other studies in Kenya (2.41%) [40] and India (2.63%) [36].

Ampicillin and aminoglycosides (mainly gentamicin) are the first-line empirical antibiotics used in our NICUs. Quinolones (ciprofloxacin) are not recommended for use in young children. However, they may be used in culture-proven sepsis with bacteria resistant to other antibiotics. For this reason such sensitivities were tested.

Among the Gram-negative isolates, all* Klebsiella pneumoniae*,* Serratia marcescens*, and* Acinetobacter baumannii* isolates were resistant to ampicillin, amoxicillin-clavulanic acid, cefotaxime, and ceftriaxone. Ceftazidime was only effective against 30% of all* Serratia* isolates.

Aminoglycosides, gentamicin and amikacin, had an intermediate effect on* Klebsiella* isolates. Similar results were observed for gentamicin against* Serratia* isolates. In contrast, amikacin had no effect against* Serratia* isolates.

The best sensitivity was observed with imipenem and quinolones, which varied from complete sensitivity by all* Serratia* isolates to lower level by* Klebsiella* isolates. Despite the high resistance of* Acinetobacter* isolates to quinolones including all strains, 29.57% of these isolates were sensitive to imipenem.

The resistance of all* Klebsiella* isolates to ampicillin was previously reported [60]. In addition, in another study in Iran, all* Klebsiella* isolates from neonates were resistant to ampicillin, while 31%, 46%, and 27% were resistant to ceftriaxone, amikacin, and gentamicin, respectively [53].

In our study, only two isolates of* Pseudomonas aeruginosa* were recovered from blood cultures exhibiting resistance to all antibiotics tested in this study.

In our study, all CoNS isolates showed high resistance to ampicillin and oxacillin.* S. haemolyticus *isolates had the highest level of resistance (≥85%) among the other CoNS isolates to amoxicillin-clavulanic acid, cefotaxime, ceftriaxone, erythromycin, azithromycin, and gentamicin. These results are in agreement with previous studies [61, 62]. Amikacin was effective against 69.70% of* S. epidermidis* isolates and all of* S. haemolyticus* and* S. hominis *isolates. The sensitivity of different CoNS spp. to amikacin, imipenem, and quinolones was variable.* S. epidermidis* was highly sensitive to imipenem, followed by quinolones, then amikacin.* S. haemolyticus* was 100% sensitive to amikacin, followed by imipenem. Concerning* S. hominis* isolates, these strains were all sensitive to amikacin and imipenem, while exhibiting lower activity to quinolones.

Interestingly, all staphylococcal isolates were sensitive to vancomycin as previously found in other reports [35, 46, 63], but its overprescription may result in the development of vancomycin-resistant strains such as enterococci.

According to our finding, best sensitivity among Gram-negative isolates was observed with imipenem followed by quinolones, while among Gram-positive isolates, vancomycin is followed by imipenem, amikacin, and finally quinolones.

## 5. Conclusion

Appropriate identification of the sepsis source, prompt antibiotic prescription, and aggressive management can effectively prevent adverse events following neonatal sepsis. Determination of the neonatal sepsis incidence, causative pathogens, and the patterns and rates of antibiotic resistance among all the neonate and infant populations are necessary to prevent complications.

## Figures and Tables

**Table 1:** Age and sex distribution among 344 neonates with suspected sepsis at Mansoura hospitals.

	Category			*P* value
	Neonates with EOS (≤72 hr) number (%)	Neonates with LOS (>72 hr) number (%)	Total number (%)	
Total	152 (44.19)	192 (55.81)	344	
Blood culture results				
Proven sepsis	49 (32.2)	91 (47.4)	140 (40.7)	0.0063 (<0.05)
Possible sepsis	103 (67.8)	101 (52.6)	204 (59.3)	
Sex				
Male	93 (61.2)	102 (53.1)	195 (56.7)	0.1650 (>0.05)
Female	59 (38.8)	90 (46.9)	149 (43.3)	

EOS: early-onset sepsis, LOS: late-onset sepsis.

**Table 2:** Maternal and neonatal data of the 304 neonates investigated for sepsis at Mansoura hospitals.

Characteristics	Total (*n* = 304) number (%)
(I) Maternal data	
Gestational age	
≤33 weeks (preterm)	108 (35.5)
34–36 weeks (late preterm)	71 (23.4)
≥37 weeks (term)	125 (41.1)
Place of delivery	
Hospital	230 (75.7)
Clinic	66 (21.7)
Home	8 (2.6)
Mode of delivery	
Vaginal	92 (30.3)
Caesarean section	212 (69.7)

(II) Neonatal data	
Weight at birth	
≤1000 g (VLBW)	21 (6.9)
1001–1500 g (VLBW)	60 (19.7)
1501–2500 g (LBW)	131 (43.1)
>2500 g	92 (30.3)

VLBW: very low birth weight, LBW: low birth weight.

**Table 3:** Clinical signs/accompanied diagnoses among neonates with suspected sepsis at Mansoura hospitals.

Clinical signs/accompanied diagnoses	Total (*n* = 304)
Respiratory distress	142
Pneumonia	24
Temperature instability	7
Convulsions	5
Hypoglycemia	4
Fetal distress	4
Meningitis	9
Surgical problems	
Diaphragmatic hernia without obstruction or gangrene	3
Esophageal atresia/TEF	11
Choanal atresia	1
Gastroschisis	1
Obstruction of duodenum	1
Congenital heart disease	11
Diseases of genitourinary	5
Cardiovascular collapse (shock)	3
Hematological symptoms (purpura/DIC)	2
Hypotonia/poor activities	4
Neonatal jaundice	15
Septic arthritis	1

The neonate could have more than one of the above clinical findings.

**Table 4:** Microbiological profile found in positive blood cultures from neonates with early- and late-onset sepsis.

Isolated microorganism	Total (%)	EoNS number (%)	LoNS number (%)
Gram-positive bacteria	82 (58.57)	34 (69.39)	48 (52.75)
*Staphylococcus aureus *	3 (2.14)	—	3 (3.30)
Coagulase negative staphylococci	74 (52.86)	32 (65.31)	42 (46.15)
*Streptococcus pneumoniae *	1 (0.71)	—	1 (1.10)
*Enterococcus faecalis *	2 (1.43)	2 (4.08)	—
*Micrococci *spp.	2 (1.43)	—	2 (2.20)
Gram-negative bacteria	54 (38.57)	15 (30.61)	39 (42.86)
Enterobacteriaceae			
*Escherichia coli * ^*∗*^	4 (2.86)	3 (6.12)^*∗*^	1 (1.10)
*Klebsiella pneumoniae *	20 (14.29)	2 (4.08)	18 (19.78)
*Klebsiella oxytoca *	1 (0.71)	—	1 (1.10)
*Raoultella* spp.	2 (1.43)	1 (2.04)	1 (1.10)
*Enterobacter cloacae *	1 (0.71)	—	1 (1.10)
*Citrobacter freundii *	1 (0.71)	1 (2.04)	—
*Serratia marcescens *	10 (7.14)	3 (6.12)	7 (7.69)
Other Gram-negative bacilli			
*Acinetobacter baumannii *	7 (5.00)	4 (8.16)	3 (3.30)
*Pseudomonas aeruginosa *	2 (1.43)	—	2 (2.20)
Not identified	6	1	5
Fungi			
*Candida* spp.	4 (2.86)	—	4 (4.40)
Total	140	49 (34.75)	91 (65.00)

^*∗*^Two isolates of *E. coli* were metabolically inactive *E. coli*.

**Table 5:** Distribution of bacterial isolates according to the global sensitivities.

Antibiotics	Global resistances (%)	Gram-positive cocci resistances (%) (*n* = 73)	Gram-negative resistances (%) (*n* = 54)
Ampicillin	122 (96.06)	70 (95.89)	52 (96.30)
Oxacillin	—	64 (87.67)	NT
Amoxicillin-clavulanic acid	86 (67.72)	37 (50.68)	49 (90.74)
Cefoxitin	106 (83.46)	64 (87.67)	42 (77.78)
Cefotaxime	92 (72.44)	43 (58.90)	49 (90.74)
Ceftriaxone	92 (72.44)	43 (58.90)	49 (90.74)
Ceftazidime	115 (90.55)	69 (94.52)	46 (85.19)
Imipenem	28 (22.05)	12 (16.44)	16 (29.63)

Vancomycin	—	0 (0)	NT

Gentamicin	78 (61.42)	42 (57.53)	36 (66.67)
Amikacin	50 (39.37)	13 (17.81)	37 (68.52)

Erythromycin	—	45 (61.64)	NT
Azithromycin	—	45 (61.64)	NT

Ciprofloxacin	43 (33.86)	22 (30.14)	21 (38.89)
Norfloxacin	44 (34.65)	22 (30.14)	22 (40.74)

NT: not tested.

**Table 6:** Comparative resistance percentage of Gram-negative bacteria to different antimicrobial agents.

Etiologic agents		Beta-lactams							Amino-glycosides		Quinolones	
		Penicillins		Cephalosporins				Carbapenem				
		AMP	AMC	FOX	CTX	CRO	CAZ	IPM	CN	AK	CIP	NOR
*Klebsiella species *	*n* = 21	100	95.24	66.67	95.24	95.24	95.24	23.81	61.90	66.67	38.10	42.86
*Serratia marcescens *	*n* = 10	100	100	80	100	100	70	0	70	100	0	0
*Acinetobacter baumannii *	*n* = 7	100	100	100	100	100	100	71.43	100	71.43	100	100
*Escherichia coli *	*n* = 4	100	50	50	75	75	75	0	50	0	25	25
*Pseudomonas aeruginosa *	*n* = 2	100	100	100	100	100	100	100	100	100	100	100
*Raoultella species *	*n* = 2	100	100	100	100	100	100	50	100	100	100	100
*Enterobacter cloacae *	*n* = 1	100	100	100	100	100	100	—^*∗*^	0	100	0	0
*Citrobacter freundii *	*n* = 1	0	0	100	0	0	0	0	0	0	0	0

^*∗*^Imipenem had an intermediate effect on this isolate.
